# A comparison of methods for the non-destructive fresh weight determination of filamentous algae for growth rate analysis and dry weight estimation

**DOI:** 10.1007/s10811-017-1157-8

**Published:** 2017-06-10

**Authors:** Michael E. Ross, Michele S. Stanley, John G. Day, Andrea J.C. Semião

**Affiliations:** 1 Institute for Infrastructure and Environment, School of Engineering, The University of Edinburgh, The King’s Buildings, Thomas Bayes Road, Edinburgh, EH9 3FG UK; 2 Scottish Association for Marine Science (SAMS), Scottish Marine Institute, Oban, Argyll PA37 1QA UK

**Keywords:** Alga, Fresh weight, Dry weight, *Cladophora* sp., *Spirogyra* sp., Growth rate

## Abstract

The determination of rates of macroalgal growth and productivity via temporal fresh weight (FW) measurements is attractive, as it does not necessitate the sacrifice of biomass. However, there is no standardised method for FW analysis; this may lead to potential discrepancies when determining growth rates or productivity and make literature comparison problematic. This study systematically assessed a variety of lab-scale methods for macroalgal FW measurement for growth rate determination. Method efficacy was assessed over a 14-day period as impact upon algal physiology, growth rate on basis of FW and dry weight (DW), nitrate removal, and maintenance of structural integrity. The choice of method is critical to both accuracy and inter-study comparability of the data generated. In this study, it was observed that the choice of protocol had an impact upon the DW yield (*P* values = 0.036–0.51). For instance, those involving regular mechanical pressing resulted in a >25% reduction in the final DW in two of the three species studied when compared to algae not subjected to any treatment. This study proposes a standardised FW determination method employing a reticulated spinner that is rapid, reliable, and non-destructive and provides an accurate growth estimation.

**Electronic supplementary material:**

The online version of this article (doi:10.1007/s10811-017-1157-8) contains supplementary material, which is available to authorized users.

## Introduction

Macroalgae encompass a phylogenetically diverse range of macroscopic plants, mainly of marine origin. They are key constituents of marine ecosystems and are a commercially and environmentally valuable natural resource. For instance, algae are renowned for their potential as a feedstock for renewable bioenergy and are already mass cultivated for food and phycocolloid industries. Furthermore, they may be grown for wastewater amelioration purposes or bio-prospected for value-added products (Fleurence 1999; Zemke-White and Ohno 1999; Hafting et al. 2012; Borowitzka 2013; Schiener et al. 2015). The increased realisation of the commercial potential of macroalgae as a direct product or as a feedstock for further processes has necessitated the optimisation of current practices and the development of a range of new tools and cultivation approaches (Griffiths et al. 2016). Furthermore, determination of the impact of abiotic and biotic conditions on biomass productivity during an experimental timeline requires the development of a set of standardised methods, which allows comparisons to be made between both treatments and experiments.

Determining algal biomass productivity through its temporal growth rate is one of the most fundamental aspects of algal research in biological, environmental, and engineering fields. For example, monitoring algal growth of taxa, including *Cladophora*, in Integrated Multi-Trophic Aquaculture (IMTA), where they perform a key bioremediation role (de Paula Silva et al. 2008), or for potential biomass applications such as bioenergy (Lawton et al. 2013), is critical to assessing both performance and productivity. Yet, there remains no standardised approach for determining growth. The primary parameters to consider when quantifying biomass are reproducibility, reliability, and applicability. Other desirable facets of a quantification method include: ease of use/speed and minimal/no damage to the biomass, where the latter issue is especially relevant for the accurate assessment of growth rates. Errors associated with determining total biomass, or growth rate, can lead to inaccuracies in estimating productivity and economic potential, as well as difficulties with literature comparison. It is therefore important to standardise procedures that are both accurate and reliable. Furthermore, the method deployed has to be applicable for the species being studied as macroalgae, and algae, in general, have varied phenotypes and growth habits. These characteristics effectively dictate the approach that may be applicable. In most cases, this is straightforward: for instance, many micro-algal taxa are unicellular and their growth can be quantified by counting the cells in a given volume of water, e.g. using either a haemocytometer, a Coulter counter (Guillard and Sieracki 2005; Marie et al. 2005), or alternatively by methods employing absorbance (Das et al. 2011) or light scattering (Yamaoka et al. 1978). For multicellular algae, these approaches are unsatisfactory as optical methods require a uniform suspension of material so that a linear relationship with biomass (weight or cell number) may be determined. In contrast, for large species of seaweed, such as members of the Lamiariales, changes in biomass can be determined by temporally measuring the length of the fronds, which can reach more than 60 m in length (Kain 1982; Bold and Wynne 1985; Dean and Jacobsen 1986; Hepburn and Hurd 2005). However, the morphology of the thalli of some macroalgal species can be quite varied, ranging from simple blades to more structurally complex forms made up of parenchyma and corticated filaments (Hurd et al. 2014). Therefore, determining the biomass of species with variegated or multifarious thalli can be complex.

A commonly employed method to determine growth rate is based on the dry weight (DW) of the organism, usually achieved by drying in an oven, freeze drier, or by the sun (Mata et al. 2010; Sharma et al. 2013). Although this approach is reliable, simple, and reproducible, the drawback is that it involves sacrificing the whole of the biomass sample, making the determination of growth rates impossible over a time course. When assessing temporal growth using DW, the problem of sacrificing samples can be overcome by utilising multiple replicates. However, this approach has its own constraints and pitfalls such as the time taken to ensure the sample has fully dried, a requirement for a large working area and other resources, as well as potential limitations in availability of biological material where sacrificing material would compromise the accuracy of the experiment.

Image analysis is a possible option as a method to determine yields and growth rates. This approach has previously been successfully applied to macroalgae where individual *Cladophora* filaments on agar have been measured temporally using light microscopy (de Paula Silva et al. 2008). However, from a practicality perspective, this approach is better suited for screening projects involving individual filaments. Macleod et al. (2016) used an alternative imaging software for the analysis of biofouling coverage on buoys as proxies for renewable energy structures in the marine environment. This approach, although simple and time efficient, could only provide data on coverage and not on the biomass of the adhering flora and fauna. Although imaging software is becoming a lot more powerful, making these techniques more readily applicable, there are still constraints including the time and resources required for analysis. Additionally, the three-dimensional and often fractal nature of seaweed makes determination of any correlation between each image and its corresponding DW, or productivity, challenging.

In many environmental and applied studies, both mass and growth rates of macroalgae are expressed as fresh weight (FW) (Gordon and McComb 1989; Peckol and Rivers 1995; Rivers and Peckol 1995). Despite the fact that FW is widely assessed, no standardised method has been agreed and thus comparisons between different studies are challenging. For instance, the FW of the chlorophyte *Cladophora* has been determined employing a variety of approaches, the most common of which is drying with a sorbent material, i.e. filter paper (Robinson and Hawkes 1986; Planas et al. 1996; Pinowska 2002; Lamai et al. 2005). However, variations in material used, application time and pressure will inevitably lead to differences in the volume of water removed. In some studies, FW is mentioned but no method of determination is reported (Ozimek et al. 1991; Choo et al. 2004; Lawton et al. 2013). On this basis, it may not be feasible, or valid, to draw conclusive inferences when comparing data from different studies.

Another key consideration is the morphology of algal species. Filamentous algae are multicellular, multifarious, and often quite fragile (Robinson 1983), which makes accurate growth rate and biomass quantification problematic. If the viability of the algae is impaired during fresh weight quantification, for instance due to excessive pressure or dehydration, this may have major implications on the accuracy of any assessments of subsequent growth. A promising, yet seldom employed method, with seemingly low mechanical impact, involves dewatering filamentous algae using a reticulated spinner (RS). In their respective in situ studies on ecology and IMTA, both Peckol et al. (1994) (Peckol and Rivers 1995) and de Paula Silva et al. (2008; 2012) employed this approach to remove excess water from *Cladophora*. However, these studies did not detail the number or duration of iterations with the reticulated spinner, thus making a comparison difficult due to the possibility of non-standardisation in approach. Furthermore, FW was only assessed at the beginning and end of each experiment lasting 10–14 days, and the daily growth rate inferred from the two data points. This non-intrusive method could have the potential to be used periodically during an experiment to determine growth rate, with little-to-no physiological detriment to the organism, thus providing a higher degree of resolution to productivity data.

The ability to accurately determine FW and productivity is the cornerstone of any algal research, and the development and use of a robust, standardised lab-scale method is an absolute necessity. This study aimed to investigate the suitability of a variety of methods for the FW determination of filamentous macroalgae employing model strains of *Cladophora* and *Spirogyra*. This is the first study of its kind to make a concerted effort to assess FW methodologies in terms of reliability and reproducibility, as well as their biological impact in terms of viability, growth, and nutrient uptake. Furthermore, the objective was to adopt a dewatering technique that is a good indicator of DW and has no detrimental impact upon the algae over a time course, therefore maintaining their original experimental purpose.

## Materials and methods

### Macroalgal strains and culture conditions

The algal strains studied were obtained from the Culture Collection of Algae and Protozoa (CCAP), at the Scottish Association for Marine Science (SAMS, Oban, UK). These included two marine isolates: *Cladophora coelothrix* CCAP 505/10, *Cladophora parriaudii* CCAP 505/09, and the freshwater isolate *Spirogyra varians* CCAP 678/3 (see http://www.ccap.ac.uk/our-cultures.htm).

The marine taxa were cultivated in 250 mL Guillard’s F/2 medium (see http://www.ccap.ac.uk/pdfrecipes.htm), based on artificial seawater at 33.5 g L^−1^ (Instant Ocean, Nemo’s World, UK) (Guillard and Ryther 1962). The freshwater isolate *S. varians* was grown in 250 mL of Jaworski’s Medium (see http://www.ccap.ac.uk/pdfrecipes.htm). The cultures were incubated in an illuminated shaker (Sartorius Stedim Biotech, Germany) at 24 °C, under an 18:6 h (light/dark) photoperiod with 30–40 μmol photons m^−2^ s^−1^ of photosynthetically active radiation (PAR 400–700 nm) (LM-100 Light Meter, Amprobe, Germany) at 100 rpm. After a 7-day acclimation period, 35.7 mg FW sub-samples determined employing the reticulated spinner, beaker + reticulated spinner (B+RS) method described in Table 1, were inoculated into triplicate 100 mL flasks containing 50 mL of the experimental media and then incubated as outlined above for 14 days. Samples were aseptically removed in a laminar flow five times over the 14-day growth period (MSC Advantage, Thermo Scientific) for FW and nutrient determination.

**Table 1:** A description of methods used for fresh weight (FW) determination and their acronyms

Method employed	Abbreviation	Description of procedure
Beaker	B	Using a spatula, algal biomass was transferred directly from the flask to a weigh boat and weighed gravimetrically.
Beaker + reticulated spinner	B+RS	B, followed by reticulated spinner (RS) centrifugation with optimised time (see below) and then weighed gravimetrically.
Beaker + filter paper	B+FP	B, followed by gently pressing the biomass with GF/F filter paper (FP) and then weighed gravimetrically.
B + reticulated spinner + filter paper	B+RS+FP	B+RS, followed by gentle pressing of the biomass with GF/F filter paper and then weighed gravimetrically.
Beaker + cavity microscope slide^a^	B+MS^a^	B, followed by placing the biomass between two cavity microscope slides (MS) to remove excess water and then weighed gravimetrically.
Perforated crucible^a^	PC^a^	Cultures were poured through a perforated crucible (PC) (Coors Gooch crucible) and then weighed gravimetrically.
Perforated crucible + reticulated spinner^a^	PC+RS^a^	PC, followed by reticulated spinner centrifugation with optimised time (see section below) and then weighed gravimetrically.
Positive control	+ C	The positive control was only weighed at the end of the experiment. Therefore, it remained unperturbed during the experimental period.

^a^Employed with *S. varians*

### Fresh weight determination

Seven different techniques for algal FW determination were assessed in this study. The different biomass dewatering methods involved centrifugation with a reticulated spinner, gently blotting with filter paper, agglomeration using a perforated crucible, and pressing between microscope slides or a combination of the above. The methods used are described in detail in Table 1. During the 14-day incubation period, the algal biomass was removed a total of five times and the different methods applied, followed by gravimetrical weighing with an analytical balance (PS-60, Fisher Brand, UK) for FW determination. After each assessment, the algal samples were transferred back to their original flasks and returned to the standardised cultivation regime.

### Optimisation of reticulated spinner FW determination

Some of the methods tested removed excess water from algal biomass by centrifugation using a small Chef’n Salad Spinner (Electronic Supplementary Material), referred to from hereon as reticulated spinner (RS). This operates using a lever, which, when pressed, rotates an internal basket. The basket has a diameter of 370 mm, with elliptical or circular perforations of maximal and minimal sizes of 18.5 mm × 3 mm to 3 mm × 3 mm, respectively. The optimal duration of dehydration using the reticulated spinner was determined for all algal species. Initially, samples were measured using the B method, as described in Table 1, and then sequentially spun in 15 s intervals, for a total of 120 s, with the FW determined after each step by weighing using an analytical balance (PS-60, Fisher Brand, UK).

### Dry weight determination

After 14 days, all algal samples were harvested and rinsed with deionised water to remove extracellular salts and nutrients. Excess water was removed using the B+RS method for *C. coelothrix* and *C. parriaudii* or the perforated crucible + reticulated spinner (PC+RS) method for *S. varians* and samples frozen (Table 1). The frozen algal biomass was then freeze-dried overnight (Modulyo 4K freeze dryer), or until a <5% variation in final mass was achieved. The lyophilised biomass was weighed gravimetrically using an analytical balance (PS-60, Fisher Brand, UK) to determine its DW.

### Microscopy

The effect of the procedures on gross cellular morphology was examined using an inverted microscope (Eclipse TE2000-U, Nikon, UK). After 14 days, samples were mounted on a microscope slide with a small volume of growth medium, to avoid desiccation. Filaments on the periphery of the culture were selected for ease of visualisation and were examined under a 100× objective lens. Images were captured using a CoolSNAP HQ2 camera (Photometrics) assisted by MetaMorph® Microscopy Automation and Image Analysis Software (Molecular Devices).

### Residual nutrient determination

The concentration of nitrate in the culture media was measured for each of the five sampling days, as well as day 0. Soluble nitrate was measured by ion chromatography (883 Basic IC Plus, Metrohm, UK), equipped with a peristaltic pump, an 863 Compact Autosampler, a Metrohm A sup 5250/4.0 mm column, and a 850 Professional IC conductivity detector. The eluent employed was 3.2 mM sodium carbonate and 1 mM sodium bicarbonate per L of dH_2_O. A MSM Suppressor, operated at 10 MPa, was used to suppress the eluent, using 0.1 M H_2_SO_4_, 0.1 M oxalic acid, and 5% (*v*/*v*) acetone per L of dH_2_O as the regenerant. Blanks and internal standards were analysed periodically to ensure the accuracy of the method.

### Optimised method—validation of the temporal FW/DW relationship

To ensure that FW growth rates determined with the optimal method from Table 1 were an accurate measurement of biomass growth, the constancy of the relationship between FW and DW growth rates was determined. A total of 15 flasks of each algal species were inoculated and incubated under the standard regime as outlined above. The algal biomass in these flasks was harvested on days 0, 3, 5, 10, and 14 following the B+RS method for *Cladophora* sp. or the PC+RS method for *S. varians* (Table 1) and the FW determined gravimetrically using an analytical balance (PS-60, Fisher Brand, UK). Three flasks of each algal species were subsequently sacrificed for the determination of their DW, as outlined above.

Growth rates for FW and DW were determined according to the formula prescribed by Yong et al. (2013):

$$ \mathrm{Growth}\ \mathrm{Rate}\left(\%\right)=\left[{\left(\frac{W_{\mathrm{t}}}{W_0}\right)}^{1/\mathrm{d}}-1\right]\times 100 $$

where *W*_t_ and W_0_ is the final and initial mass and *d* is the time (days).

### Statistical analysis

All experiments were performed in triplicate and the experimental error was calculated and expressed as one standard deviation (SD). The significance of difference in the DW yield of macroalgal samples periodically subjected to a variety of dewatering methods was obtained by one-way ANOVA with Tukey’s post hoc analysis (*P* = <0.05; *n* = 3). Pearson correlation coefficients, *r*, were used to assess the temporal relationship between FW and DW. All statistical analysis was performed using Minitab Statistical Software version 17.

## Results and discussion

In this study, a variety of methods, described in Table 1, were assessed for the determination of the FW of three species of filamentous macroalgae: *C. coelothrix*, *C. parriaudii*, and *S. varians* (Electronic Supplementary Material). These three species have differing physical appearances and growth characteristics. *Cladophora coelothrix* grows quite slowly in tightly knit “clusters” with thick cell walls, whereas *C. parriaudii* grows quickly in a loose skein. *Cladophora* has been described as an “ecological engineer”: they are a robust, bloom-forming species and have shown high removal rates of nutrients and heavy metals (Deng et al. 2006; Deng et al. 2009; de Paula Silva et al. 2012; Zulkifly et al. 2013; Liu and Vyverman 2015). Furthermore, they are resistant to grazers (Zulkifly et al. 2013), making them strong candidate species for wastewater bioremediation (de Paula Silva et al. 2012). *Spirogyra varians* has a central core of biomass, from which helical-shaped filaments grow toward the water surface. These filaments are very fragile, tending to fragment when disturbed.

The three species were selected as model organisms to explore the applicability of dewatering methods across a range of phenotypes. Systematic measurement of FW, final DW, FW/DW ratio, and NO_3_^−^ uptake and microscopic image analysis were used to ascertain the viability, growth, and metabolic activity of the algae periodically subjected to the different harvesting methods.

### Optimisation of the reticulated spinner

**Fig. 1:**
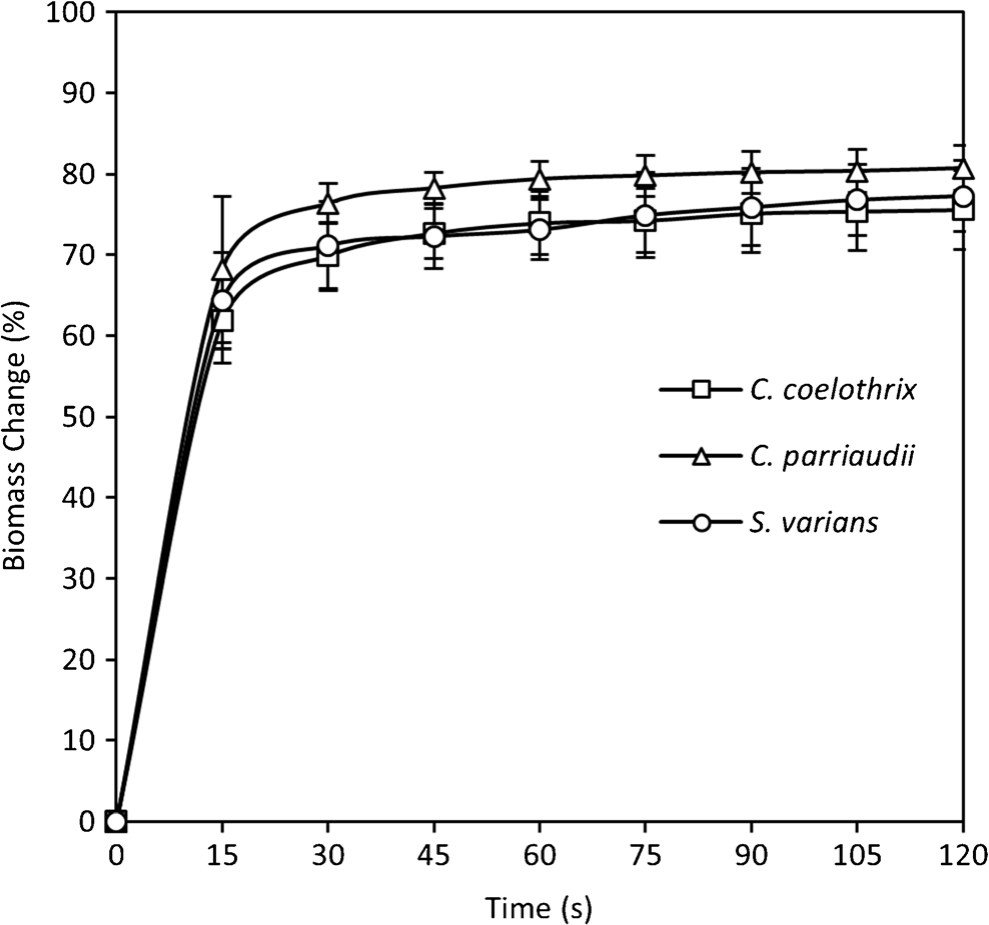
Optimisation of spinning time required to dewater *C. parriaudii*, *C. coelothrix*, and *S. varians* in the reticulated spinner. Algal FW values were measured after 15 s increments in the reticulated spinner. The biomass change (%) represents the mass change of FW relative to the initial wet biomass (*n* = 3, *error bars* denote 1 SD)

Some of the harvesting methods tested employ a reticulated spinner, which has the ability to rapidly remove extracellular water from filamentous algae and hence facilitate accurate FW determination. In order to ensure a consistent level of water removal, the operation of the reticulated spinner was standardised. The FW of the three algal species was determined after each 15 s spin, up to a maximum duration of 120 s (Fig. 1). There was a reduction in the overall weight corresponding to 77–81% of the original wet weight, irrespective of the species studied. This indicated the potential applicability of the method to a wide range of filamentous taxa. The majority of water removal, i.e. 61–68%, occurred within the first 15 s. This was followed by a reduction in the rate of weight change, with minimal further water removal after 90 s operation, corresponding to a reduction in mass up to 75–80%. Additional spinning, beyond 90 s, resulted in a further reduction in mass of less than 1.5% for all species tested. A spinning time of 90 s was adopted for the reticulated spinner. It is recommended that a similar approach is employed when implementing and standardising this method for different algal taxa, varying amounts of algal biomass, or when cultivating in very different conditions, such as extremes of salinity (Angell et al. 2015).

### Dewatering efficiency for the tested methods

**Fig. 2:**
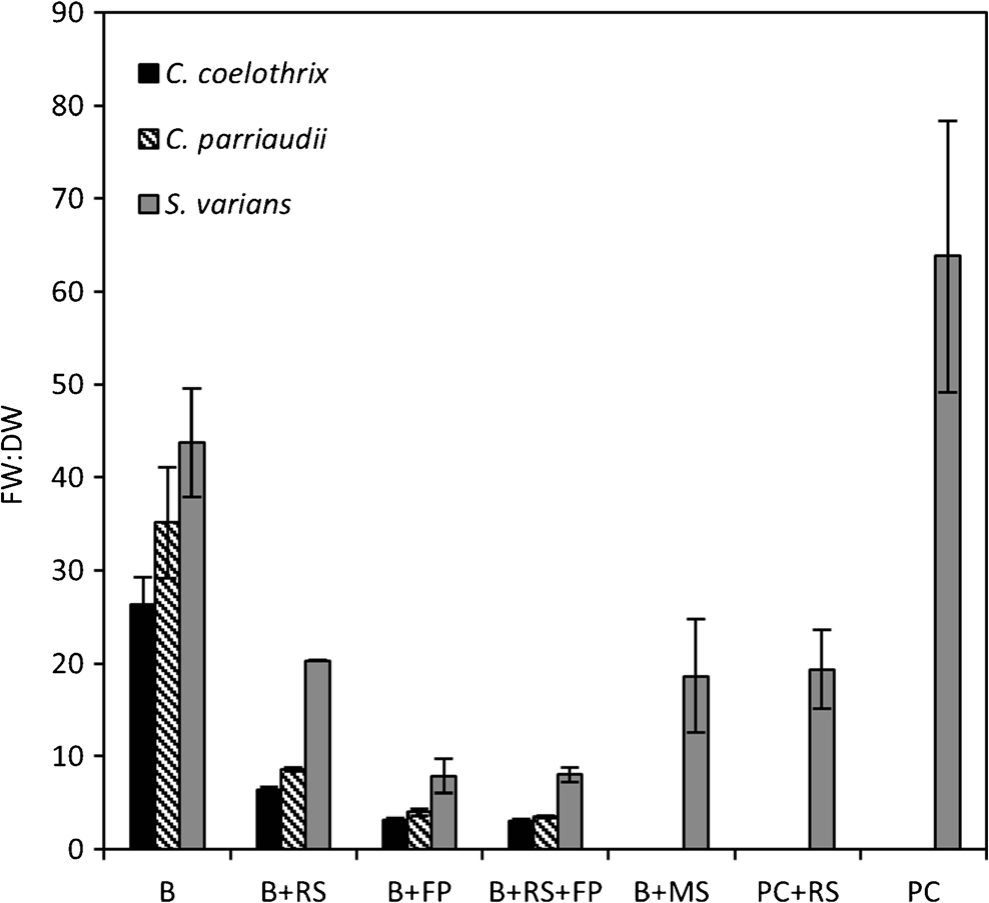
Final fresh weight to dry weight ratio of *C. parriaudii*, *C. coelothrix*, and *S. varians*, grown for 14 days (100 rpm, 24 °C, light intensity of 30–40 μmol photons m^−2^ s^−1^, 18:6 h L/D photoperiod), periodically harvested, and dewatered following the methods: beaker (*B*), beaker + reticulated spinner (*B+RS*), beaker + filter paper (*B+FP*), beaker + reticulated spinner + filter paper (*B+RS+FP*), beaker + cavity microscope slide (*B+MS*), perforated crucible + reticulated spinner (*PC+RS*), and perforated crucible (*PC*). More detailed descriptions on each method can be found in Table 1 (*n* = 3, *error bars* denote 1 SD)

Although DW is an accurate measure of biomass, its determination necessitates the sacrifice of the culture. However, FW determination is non-destructive and can be reiterated across a time series to give high-resolution productivity data. In this study, cultures were harvested periodically over a 14-day period to obtain FW, and on the final day, DW was also determined and an FW/DW ratio obtained (Fig. 2). This ratio would be expected to inform how strong an indicator of biomass a particular dewatering method is: the lower the ratio, the more efficient the dewatering method should be. However, the size of the error bars will also indicate how reproducible each method is, therefore providing a more accurate and consistent measure of actual productivity.

Of the methods tested, B and PC required the least mechanical effort, but they resulted in high FW/DW ratios for the three species, ranging between 26–44 and >60, respectively for *Cladophora* sp. and *S. varians* (Fig. 2). Although one would anticipate that these methods would not result in any physical damage to the alga and therefore have no deleterious effects on metabolic function or growth, they did have a high degree of error that was associated with the greater volume of unpredictable water carry-over, making these methods unsuitable for implementation. Conversely, beaker + filter paper (B+FP) and beaker + reticulated spinner + filter paper (B+RS+FP) have FW/DW ratios of <10 for *S. varians* and <4 for both species of *Cladophora*, with low error throughout. These methods involved lightly pressing the biomass with an absorbent filter paper (Table 1) and resulted in the highest removal of water from the biomass (Fig. 2). The B+RS method, which removes water centrifugally, also has a great degree of highly consistent residual water removal, with ratios of 6.3 (± 0.3) and 8.6 (± 0.2) for *C. coelothrix* and *C. parriaudii*, respectively. Methods B+RS, beaker + cavity microscope slide (B+MS), and PC+RS all had a similar degree of water removal when employed with *S. varians*: ratios were 20.3 (± 0.05), 18.6 (± 6.1), and 19.3 (± 4.2), respectively.

**Fig. 3:**
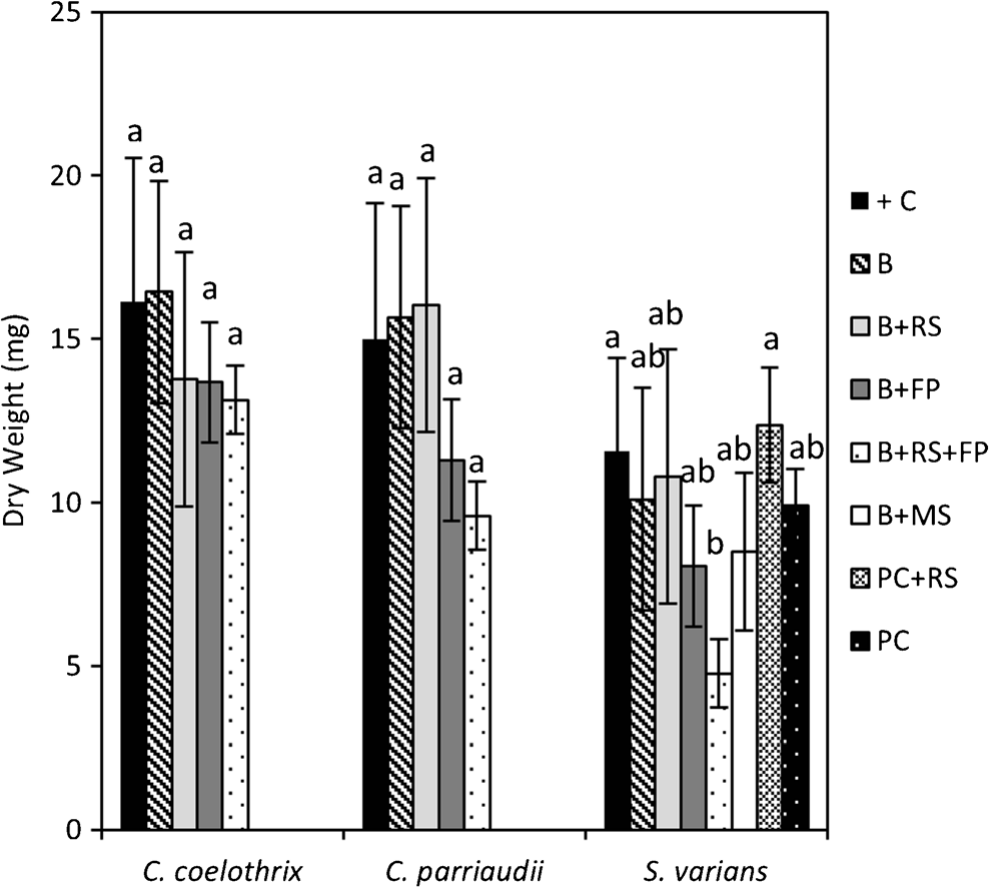
Final dry weight (*DW*) of *C. parriaudii*, *C. coelothrix*, and *S. varians*, grown for 14 days (100 rpm, 24 °C, light intensity of 30–40 μmol photons m^−2^ s^−1^, 18:6 h L/D photoperiod), periodically harvested, and dewatered following the methods: beaker (*B*), beaker + reticulated spinner (*B+RS*), beaker + filter paper (*B+FP*), beaker + reticulated spinner + filter paper (*B+RS+FP*), beaker + cavity microscope slide (*B+MS*), perforated crucible + reticulated spinner (*PC+RS*), and perforated crucible (*PC*). More detailed descriptions on each method can be found in Table 1 [*n* = 3 (except *S. varians* “+ C” *n* = 8), *error bars* denote 1 SD]. For each species, *means that do not share a letter* are significantly different from one another, *P* = <0.05

The final DW obtained for the different harvesting methods is shown in Fig. 3. The + C corresponds to biomass grown and harvested without any additional dewatering procedures being applied and hence acted as a positive control. Variations in the final DW were observed for the different methods adopted, which indicated that there was an impact on the algal growth.

The DW obtained for the different treatments of *C. coelothrix* ranged between 13.13–16.43 mg and no statistically significant differences in the yield were observed (*P* value = 0.51). This species grows in tightly knit “clusters” and has a basal cell wall thickness of up to 15 μm, which may make it resistant to mechanical damage (Leliaert and Coppejans 2003). Although the choice of FW method was not significant for *C. parriaudii* (*P* = 0.102), the DW yield was reduced from 15 mg in the positive control to 11.3 and 9.6 mg when employing methods B+FP and B+RS+FP, respectively. As shown in Table 1 and Fig. 2, methods that involve pressing the biomass with absorbent filter paper tend to have a low and reproducible FW/DW ratio, indicating good water removal. However, high levels of water removal and a low growth indicated that damage occurred during the sampling and FW determination procedures. The less dense growth habit exhibited by the *C. parriaudii* may make them more susceptible to physical damage when harsher dewatering approaches were applied. For instance, commonly used large-scale harvesting techniques, such as centrifugation and cross-flow membrane filtration, can exert large amounts of shear stress that can damage and lyse micro-algal cells (Chen et al. 2011; Bilad et al. 2013).

In the case of *S. varians*, the choice of FW method had a significant impact upon the DW yield, *P* value = 0.036. It was observed that the biomass of *S. varians* was prone to fragmentation when disturbed, and obtaining sufficient biomass to ascertain FW was problematic for several of the methods employed. For instance, disintegrating into a suspension of short filaments meant that the algae would tend to pass through the apertures of the reticulated spinner and were challenging to gently blot with a piece of filter paper. The use of a cavity microscope slide (B+MS) was intended to reduce filament loss and to minimise damage caused by actively blotting or from effects of desiccation. A pre-collection step, involving pouring the contents of the flask through a perforated crucible (PC in Table 1), was incorporated into the harvesting protocol for this alga. The apertures were small enough to retain most of the biomass and agglomerate it, allowing it to then be subjected to a further dewatering method with the reticulated spinner. As can be observed (Fig. 3), the PC method had no obvious impact on the biomass levels of *S. varians* obtained when compared to the control. However, implementing the PC step prior to utilising the RS method increased the DW yield from 10.8 to 12.4 mg, compared to employing the B+RS technique alone.

Variation in the FW/DW ratio is dependent upon the growth conditions. For instance, Angell et al. (2015) found that the FW/DW of *Ulva ohnoi* was the greatest when cultivated in low to optimal salinities and the lowest when exposed to high salinity. This difference in ratio was most likely caused by a change in osmotic potential. Care should be taken when determining FW/DW across a range of environmental variables or cultivation conditions. However, this is not the case of the present study as the algae were grown under the same conditions.

The FW/DW ratio was also found to depend upon the dewatering methods applied. Those involving spinning (B+RS, B+RS+FP, and PC+RS) or blotting with filter paper (B+FP, B+RS+FP) will result in a lower FW/DW ratio than those that apply minimal pressure, such as pouring through a perforated crucible (PC). Furthermore, the FW/DW ratio obtained and its degree of error will also depend upon the species or morphology of the alga that it is applied to. For instance, the FW/DW values varied between species using the same method due to differences in water retention, both intra- and extracellularly. Finally, the DW yield is also species specific. The choice of dewatering method will have minimal impact upon robust cultures, with thick cell walls or protective growth habits, such as *C. coelothrix*. In contrast, fragile species like *S. varians* are more strongly influenced by the choice of dewatering method, with more stringent methods compromising the viability of the culture. Furthermore, *S. varians* requires a pre-collection step to ensure the minimisation of biomass losses, which would further reduce the DW yield.

### Physiological assessment

**Fig. 4:**
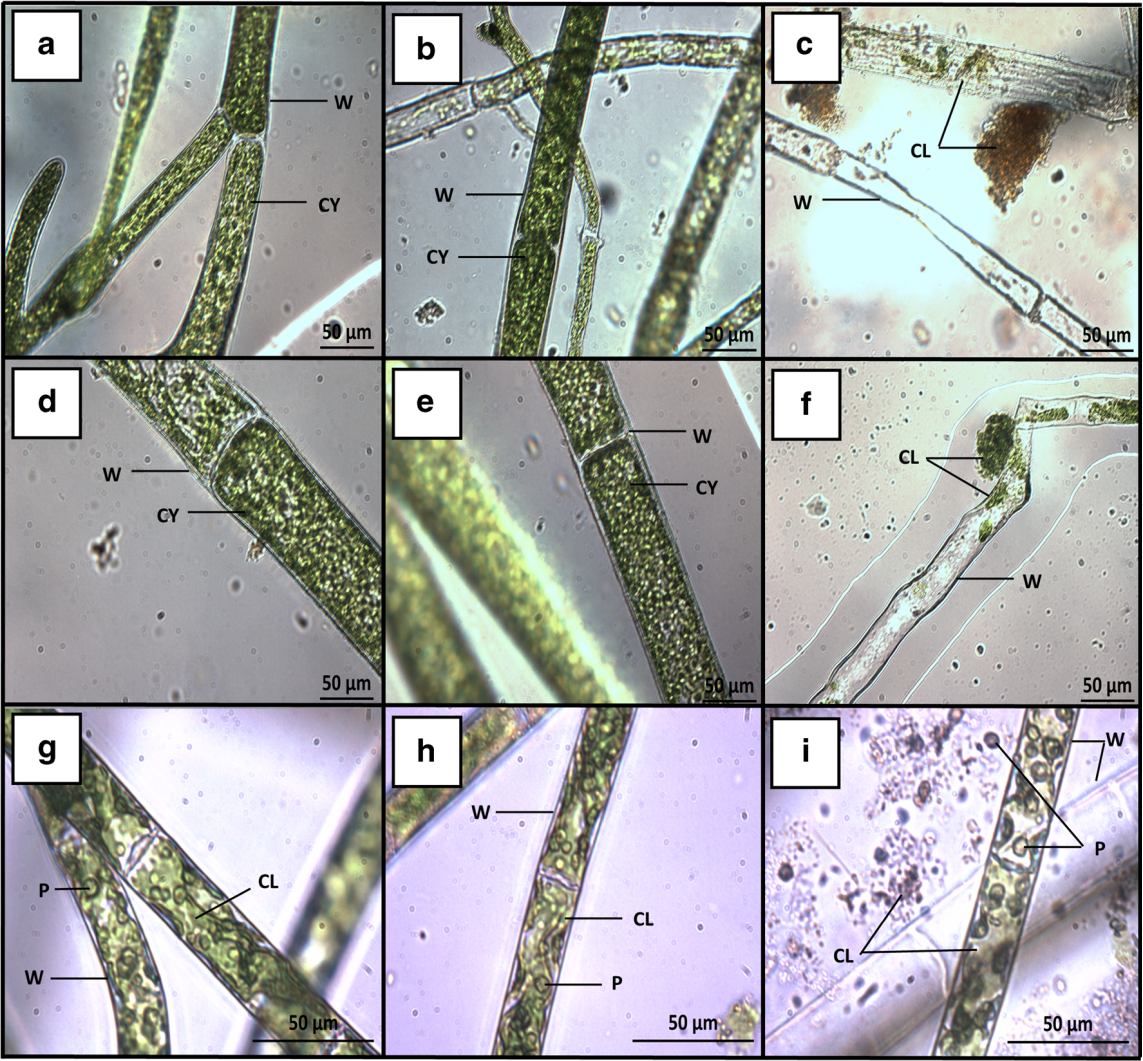
Plates of *C. coelothrix* (**a**–**c**), *C. parriaudii* (**d**–**f**), and *S. varians* (**g**–**i**) taken with an inverted microscope after a 14-day growth trial (100 rpm, 24 °C, light intensity of 30–40 μmol photons m^−2^ s^−1^, 18:6 h L/D photoperiod) with frequent harvesting using different methods described in Table 1: positive control (*+ C*) (**a**, **d**, and **g**), beaker + reticulated spinner (*B+RS*) (**b**, **e**), beaker + filter paper (*B+FP*) (**c**, **f**, and **i**), and perforated crucible + reticulated spinner (*PC+RS*) (**h**). *W* denotes the cell wall, *CL* indicates the chloroplasts, *P* is the pyrenoid, and *CY* highlights the multi-nucleate cytoplasm that contains pyrenoids, chloroplasts, and vacuoles

**Fig. 5:**
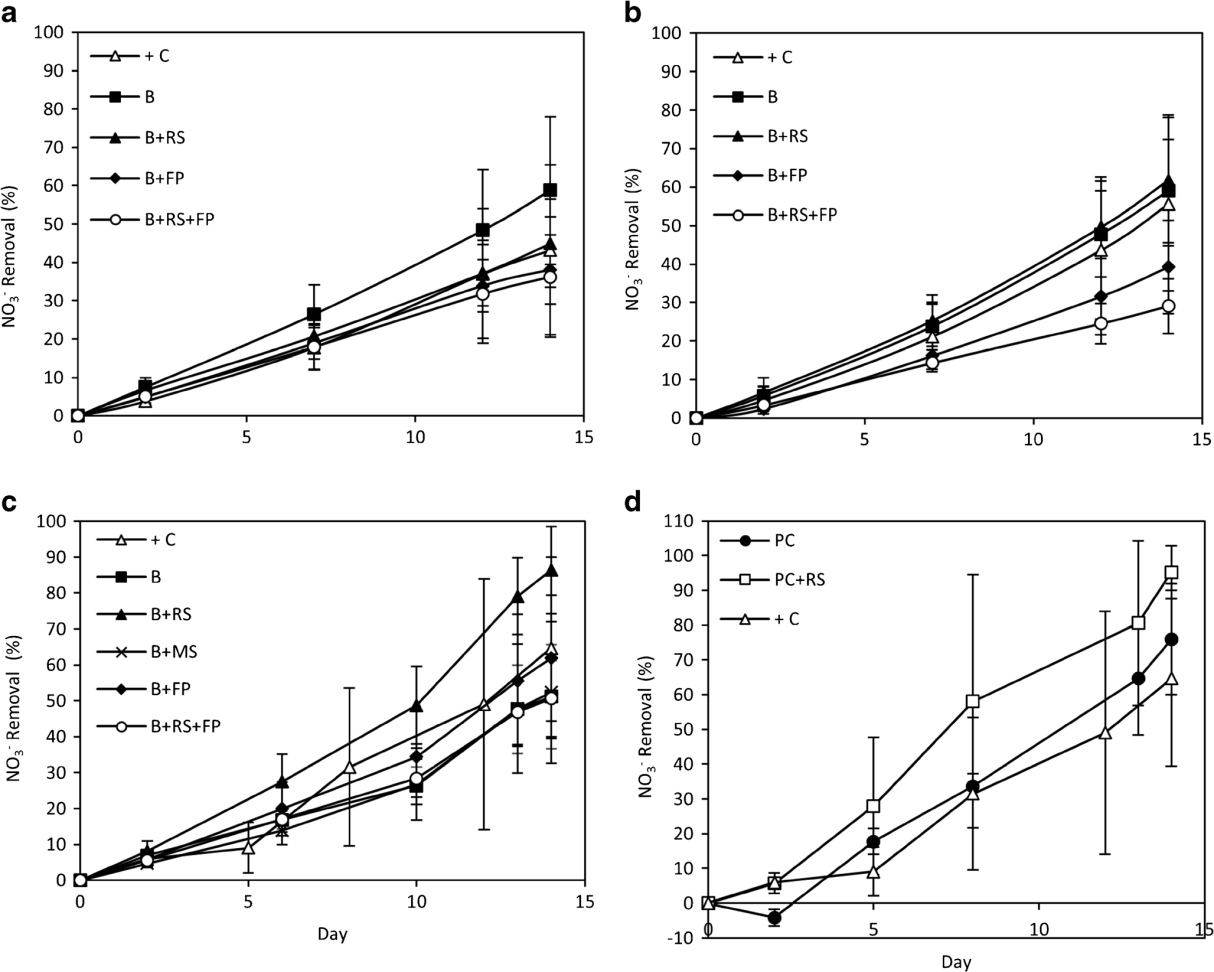
The temporal removal of nitrate from the media by *C. coelothrix* (**a**), *C. parriaudii* (**b**), and *S. varians* (**c**, **d**). Their growth was assessed periodically using different protocols: beaker (*B*), beaker + reticulated spinner (*B+RS*), beaker + filter paper (*B+FP*), beaker + reticulated spinner + filter paper (*B+RS+FP*), beaker + cavity microscope slide (*B+MS*), perforated crucible + reticulated spinner (*PC+RS*), and perforated crucible (*PC*) (Table 1). Nitrate was measured in the media using ion chromatography (*n* = 3, [except *S. varians* “+ C” where *n* = 8] *error bars* denote 1 SD)

The reduced DW yields observed for some of the species may be due to the viability of the biomass being compromised as a result of the different protocols employed. Images of the harvested algae subjected to methods + C, B+RS or PC+RS, and B+FP were taken, to ascertain whether the algae showed any physical damage (Fig. 4a–i). Healthy, undamaged filaments were observed in the positive control treatment for all three species (Fig. 4a, d, and g). The filaments were considered to be phenotypically normal as they exhibited the characteristic large breeze-block type cells, with typical green colouration throughout the cells. Furthermore, *C. coelothrix* displayed some branching, indicative of growth (Fig. 4a). *Cladophora* cultures that were periodically harvested using the B+RS method (Fig. 4 b, e) and the amended PC+RS method for *S. varians* (Fig. 5h) were similar in appearance to the positive controls, with only some superficial damage visible for *C. coelothrix*. In contrast, algal cultures periodically harvested using the B+FP treatment (Fig. 4c, f, and i) displayed obvious damage, with their cellular contents having been expelled and with chloroplasts observed in large, often discoloured conglomerates attached to the outside of the cell wall. Although the absorbent filter paper removed superficial water, it was assumed that it caused some shear or mechanical stress upon the organism in the process. The greater parity between the FW/DW ratio for methods employing a filter paper (Fig. 2) was potentially not only due to the removal of superficial and interstitial water but this approach may also have removed intercellular fluid, resulting in cellular injury, as the cells diminished in size, and were in some cases devoid of contents. The image analysis evidencing the presence or absence of mechanical or physical damage to the algal cellular morphology is in agreement with the corresponding DW data (Fig. 3). The methods employed to determine FW growth might not be appropriate as they adversely impact upon the viability of the cell. Although methods B+FP and B+RS+FP offer a good estimation of the DW yield, this comes at a cost. In addition to a reduction in the DW yield, visual imaging indicated that the B+FP technique clearly damaged all cultures tested. Given the methodological similarity between B+FP and B+RS+FP, it may be inferred that employing the B+RS+FP method results in comparable levels of cellular damage. On the other hand, B+RS and PC+RS have similar biomass yields compared to the + C for all three species, whilst providing an accurate estimation of DW and with negligible obvious damage to the algae.

Disparities in nutrient uptake were observed, depending on the FW assessment approach employed, further indicated that under some treatment regimes, physiological damage had occurred (Fig. 5). For all species tested, the positive control cultures demonstrated a high capacity to remove NO_3_^−^ from the media, with ∼45, 55, and 65% removal for *C. coelothrix*, *C. parriaudii*, and *S. varians*, respectively (Fig. 5a–d). In general, cultures subjected to the mildest dewatering methods (Table 1/Fig. 2) demonstrated the highest nitrate removal capacity: B with 59% and B+RS with 45–62% removal for both species of *Cladophora*, whereas 76 and 95% NO_3_^−^ removal was observed for *S. varians* with methods PC and PC+RS, respectively. Conversely, algae subjected to protocols that featured mechanical pressing (B+FP, B+RS+FP, and B+MS) were amongst those with the lowest rates of nutrient uptake. This indicated that the more harsh methods had a detrimental effect on the algae in terms of algal metabolism. This was further exemplified by the discrepancy in nutrient uptake between algae subjected to the FW methods after day 2, which became increasingly pronounced with each successive harvest. Conversely, in comparison with the + C, algae treated using the B+RS or PC+RS protocols had similar nutrient uptake capabilities for all species tested. This suggests that these harvesting methods have little-to-no impact on the physiological integrity of the organism. This aspect is particularly important for small-scale algal systems where routine sampling is required and sampled algae are returned to the cultivation system.

The results obtained in this study may be partially explained by the differences in morphology and algal growth strategy of the taxa studied. Members of the genus *Cladophora* are characterised by their multi-nucleate cells arranged in either branched or unbranched filaments. Their cell wall is primarily composed of highly crystalline cellulose I (Bold and Wynne 1985; Hoek et al. 1995). As previously mentioned, *C. coelothrix* (see http://www.ccap.ac.uk/our-cultures.htm) typically grows in floating clusters or mats, which are tightly wound (Electronic Supplementary Material). This characteristic provides mechanical protection to the cells and it was noted in this study that *C. coelothrix* was largely unaffected by the FW determination methods employed. In contrast, *C. parriaudii* (see http://www.ccap.ac.uk/our-cultures.htm) tends to grow in a loose skein, with filaments that grow rapidly outwards to any vacant space; this growth strategy will mean that the younger, less robust filaments are likely to be more susceptible to mechanical damage (Electronic Supplementary Material). This was observed in this study (Figs. 3, 4f, and 5b) where the DW yield, physical damage, and a reduced metabolic capability/ nutrient sequestration were observed in cultures subjected to the more stringent dewatering methods. Less mechanically stressful treatments, such as B+RS, were better suited for this species. *Spirogyra* are almost exclusively found in freshwater and are characterised by growing in unbranched filaments with an intracellular helical ribbon of chloroplasts (Whitton 1999). *Spirogyra varians* (see http://www.ccap.ac.uk/our-cultures.htm) grows as a benthic mass, with filaments intertwined in a helical arrangement growing toward the water surface (Electronic Supplementary Material). The filaments are thin, fragile, and readily fragment when agitated or swirled (Chapman and Chapman 1973; Hoek et al. 1995). This propensity for the colony to disintegrate meant that many of the approaches employed were unsuitable owing to the frailty of its filaments.

### Investigating the temporal relationship between FW/DW under optimal harvesting conditions

**Fig. 6:**
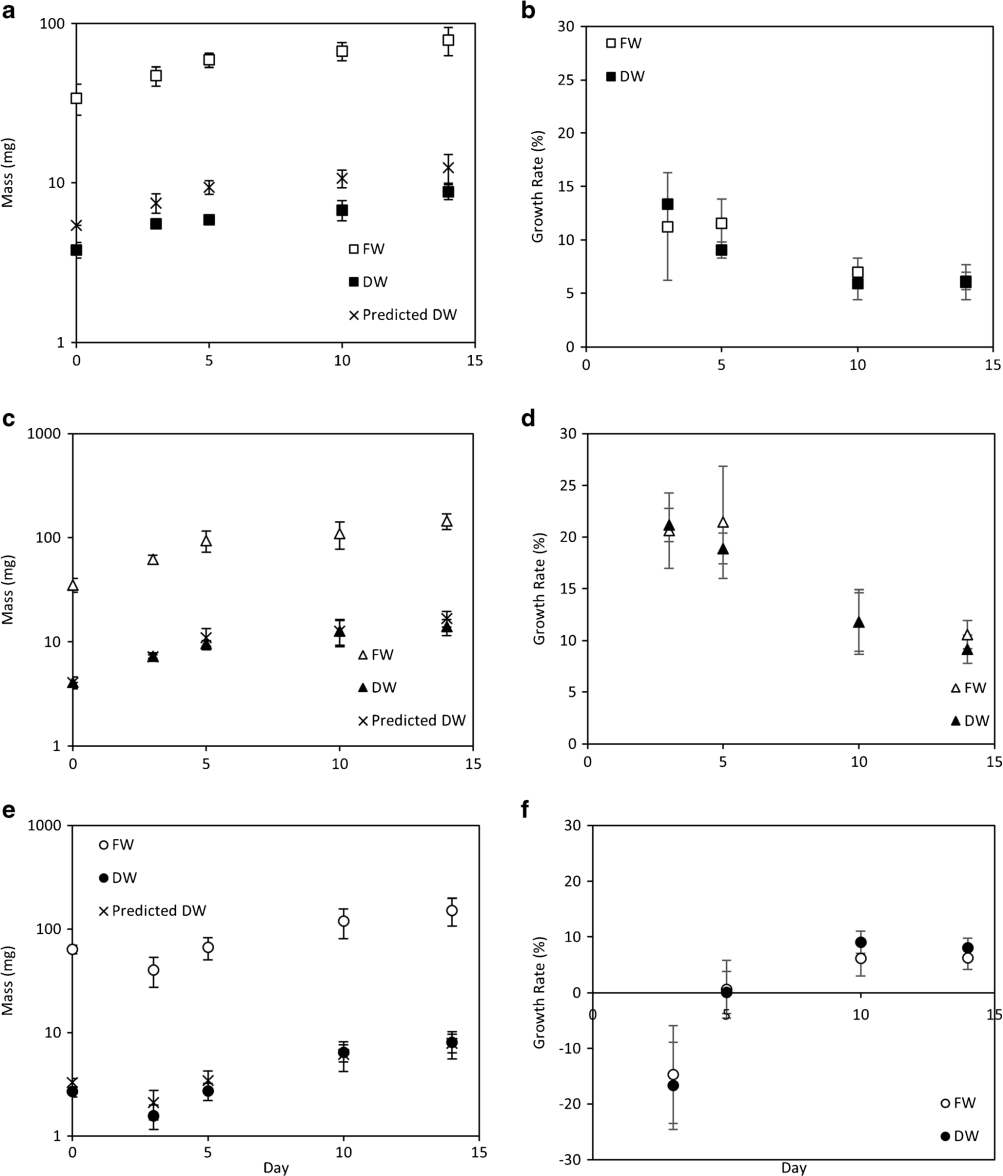
The FW, DW, predicted DW, and rates of FW and DW growth of the three species of algae: *C. coelothrix* (**a**, **b**), *C. parriaudii* (**c**, **d**), and *S. varians* (**e**, **f**). The temporal relationship between FW and DW (**a**, **c**, and **e**) was assessed using Pearson’s correlation coefficients, *r*. On each harvest day, triplicate flasks were harvested and the algal biomass was dewatered using either the optimised beaker + reticulated spinner (*B+RS*) method for *Cladophora* species or the perforated crucible + reticulated spinner technique (*PC+RS*) for *S. varians* (Table 1). The DW was attained by freezing the samples overnight followed by overnight lyophilisation (*n* = 3, *error bars* denote 1 SD)

In comparison with methods for DW determination, B+RS and PC+RS are rapid, are less energetically expensive to perform, and are non-destructive to the algal sample. In order to ensure that FW measurements using B+RS and PC+RS were reliable indicators of DW (Fig. 6a, c, and e) and consequently of biomass growth (Fig. 6b, d, and f), the relationship between FW and DW was determined for a 14-day incubation period. It was noted that there is a strong positive relationship between the FW and DW mass: Pearson correlation coefficients were determined as *r* = 0.871, 0.948, and 0.954 for *C. coelothrix*, *C. parriaudii*, and *S. varians*, respectively, with *P* values of <0.001 and with low error throughout. Interspecies variation in biomass growth rate can be clearly determined using the B+RS and PC+RS methods. The initial “dip” in growth observed for *S. varians* (Fig. 6e, f) was assumed to be caused by the fragmentation of the colony and incomplete retention of the biomass on the perforated crucible.

One of the purposes of this study was to be able to assess the feasibility of using non-destructive FW measurements to determine macroalgal growth rates, instead of using sacrificial DW measurements. The FW/DW ratios of 6.3, 8.6, and 19.3 for *C. coelothrix*, *C. parriaudii*, and *S. varians*, respectively (Fig. 2), were applied to the temporal FW measurements from Fig. 6, in order to predict the temporal DW for each species and compare against actual DW yields (all determined for identical culture conditions). This allows the accuracy of the FW method to be demonstrated. The results indicated that the B+RS or PC+RS methods can be used to estimate the DW yield of filamentous macroalgal species across time. In addition, the growth rates of FW and DW are comparable. This further demonstrated that the values are closely related and that the prescribed FW methodology can be used as a strong estimation of DW productivity.

The constancy of the FW to DW relationship, irrespective of species, backed up with statistical evidence, demonstrates that a reticulated spinner is a reliable and accurate method for generating samples for FW determination and consequently DW estimation. Moreover, the ability to accurately assess productivity between species mean this approach can be a useful tool for a variety of scientific applications, including experimental growth screening.

## Conclusions

This is the first study to systematically assess a range of dewatering approaches to determine the FW of filamentous macroalgae at lab scale using effectiveness, reliability, practicality, and biological and physical impact as factors. The results demonstrate differences in the effectiveness of a variety of dewatering methods and the physical and metabolic implications at both species and genus levels.

This study proposes a method involving a reticulated spinner that is rapid, robust, inexpensive, and easily implemented or standardised for other algal taxa or amounts of biomass. This method marries together high accuracy in biomass assessment due to excellent dewatering capabilities, with negligible impact upon algal performance, assessed as growth, nitrate removal, and structural integrity. Further studies are required for the scaling up of this method for larger cultures at pilot and full scale, which can include assessing and standardising the application of a gentle spinning cycle using a washing machine (Mata et al. 2016).

## Electronic supplementary material

Figure S1 (DOCX 2.04 mb)
