# Supplementary material for: A comparison of methods for the non-destructive fresh weight determination of filamentous algae for growth rate analysis and dry weight estimation
Source: J Appl Phycol. 2017 Jun 10;29(6):2925–36. doi: 10.1007/s10811-017-1157-8 (PMC5705739; doi:10.1007/s10811-017-1157-8)
Supplement: Supplementary file 1 — (DOCX 2.04 mb) [file 10811_2017_1157_MOESM1_ESM.docx]

A comparison of methods for the non-destructive fresh weight determination of filamentous algae for growth rate analysis and dry weight estimation: Supplementary Information

Michael E. Ross^a,b^, Michele S. Stanley^b^, John G. Day^b^, Andrea J.C. Semião^a,^^[[1]](#footnote-1)^

^a^ Institute for Infrastructure and Environment, School of Engineering, The University of Edinburgh, William Rankine Building, The King's Buildings, Thomas Bayes Road, Edinburgh, EH9 3FG, UK

^b^ Scottish Association for Marine Science (SAMS), Scottish Marine Institute, Oban, Argyll PA37 1QA, UK

E-mail addresses of authors

Mr Michael E. Ross: [m.ross@ed.ac.uk](mailto:m.ross@ed.ac.uk)

Dr Michele S. Stanley: [michele.stanley@sams.ac.uk](mailto:michele.stanley@sams.ac.uk)

Prof. John G. Day: [john.day@sams.ac.uk](mailto:john.day@sams.ac.uk)

Dr Andrea J.C. Semião: [asemiao@ed.ac.uk](mailto:asemiao@ed.ac.uk)


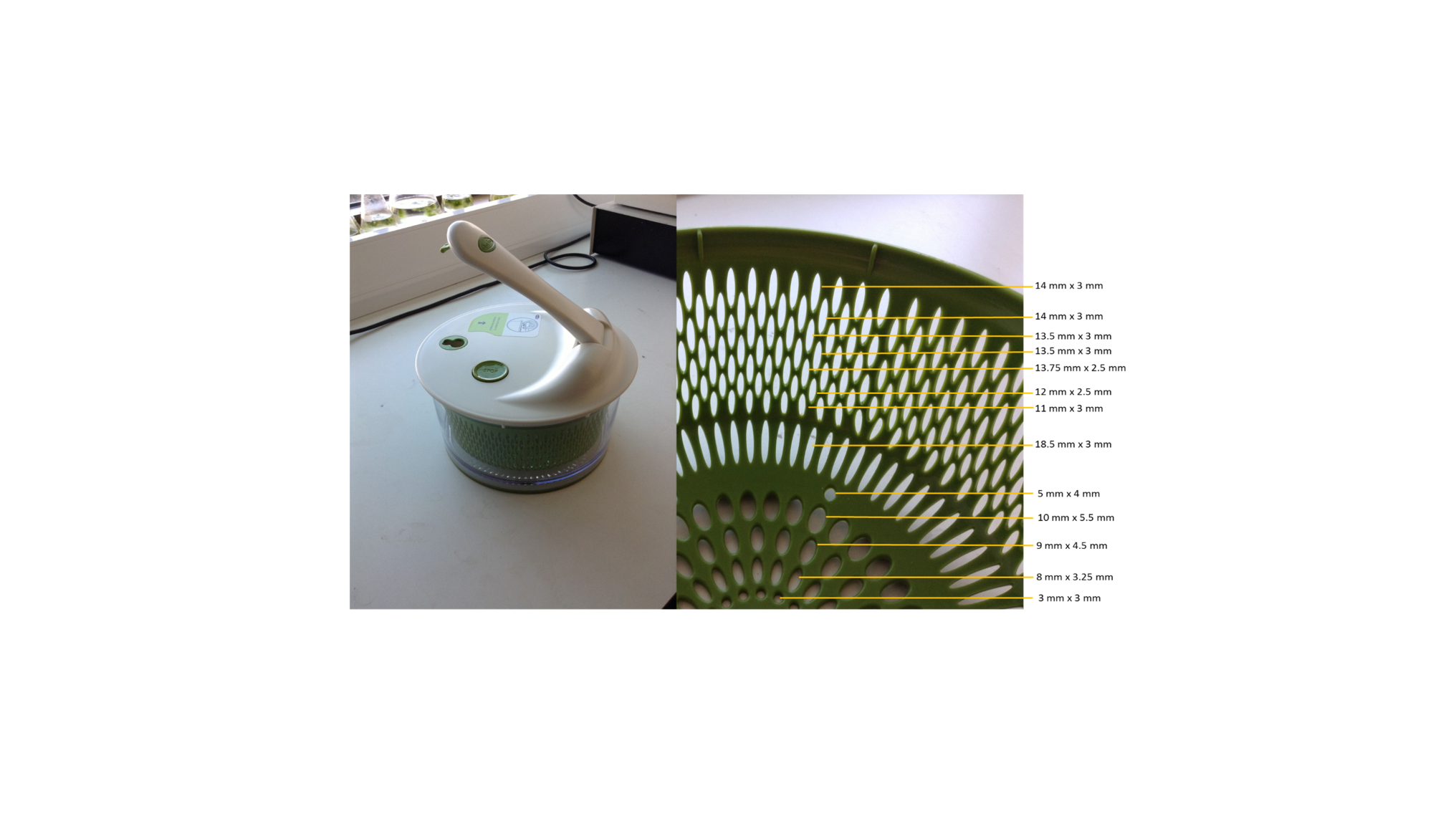


Figure S1 – Reticulated Spinner Characteristics


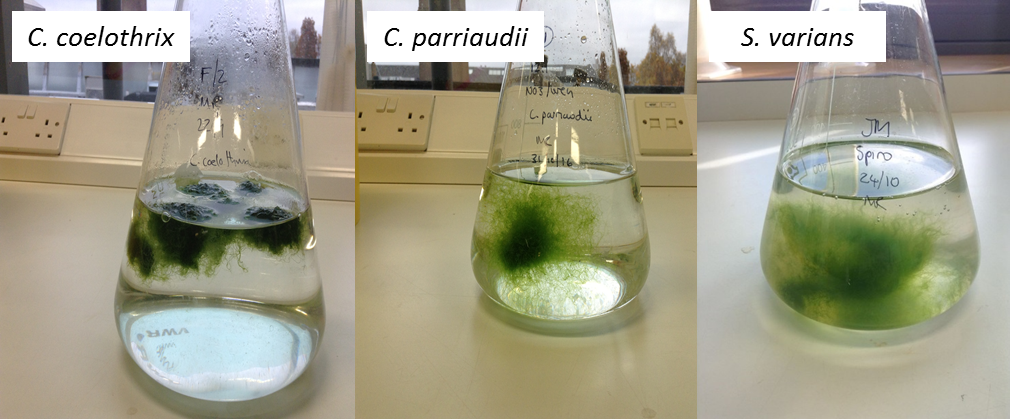


Figure S2 – Images of *Cladophora coelothrix*, *Cladophora parriaudii*, and *Spirogyra varians.*

1. **Corresponding author** (Dr Andrea J.C. Semião)**:**

   **E-mail:** [asemiao@ed.ac.uk](mailto:asemiao@ed.ac.uk)

   **Tel:** +44 (0)131 6505792

   **Fax:** +44 (0)131 506554 [↑](#footnote-ref-1)
